# Kosmotropic Anions Promote Conversion of Recombinant Prion Protein into a PrP^Sc^-Like Misfolded Form

**DOI:** 10.1371/journal.pone.0031678

**Published:** 2012-02-09

**Authors:** Rodrigo Diaz-Espinoza, Abhisek Mukherjee, Claudio Soto

**Affiliations:** 1 Department of Neurology, Mitchell Center for Alzheimer's Disease and Related Brain Disorders, University of Texas Houston Medical School, Houston, Texas, United States of America; 2 Department of Biochemistry and Molecular Biology, University of Texas Medical Branch, Galveston, Texas, United States of America; Ohio State University, United States of America

## Abstract

Prions are self-propagating proteins involved in transmissible spongiform encephalopaties in mammals. An aberrant conformation with amyloid-like features of a cell surface protein, termed prion protein (PrP), is thought to be the essential component of the infectious particle, though accessory co-factor molecules such as lipids and nucleotides may be involved. The cellular co-factors and environmental conditions implicated in PrP misfolding are not completely understood. To address this issue, several studies have been done inducing misfolding of recombinant PrP (recPrP) into classical amyloid structures using partially denaturing conditions. In this work, we report that misfolding of recPrP into PrP^Sc^-like aggregates can be induced by simply incubating the protein in the presence of kosmotropic salts at concentrations that are known to retain or increase the stability of the protein. We used a simple experimental reaction (protein, buffer and salts) submitted to agitation/incubation cycles at physiological temperature and pH. The formation of protease resistant-recPrP was time and salt-concentration dependent and required the presence of kosmotropic anions such as F^−^ or SO_4_^−2^. The molecular weights of the protease resistant recPrP fragments are reminiscent of those found in degradation assays of bona fide PrP^Sc^. The aggregates also exhibited PrP^Sc^-like ultrastructural features including rod-shape morphology under electron microscope, high beta-sheet content and thioflavin-T positive signal. The formation of recPrP aggregates with PrP^Sc^ biochemical features under conditions closer to physiological in the absence of organic co-factor molecules provides a simple setup that may prove helpful to understand the molecular mechanism of PrP misfolding.

## Introduction

Transmissible spongiphorm encephalopaties (TSEs) are fatal and infectious neurological maladies in mammals caused by prions. The infectious agent is primarily composed of an aberrantly folded glycosylated protein termed prion protein (PrP), which is mainly expressed in the brain [1], [2] . Misfolded PrP in its prion state (PrP^Sc^) acquire self-propagation features to induce the transformation of the normal PrP conformation (PrP^C^) into the infectious form [3]. In vitro experiments using brain extracts from infected and uninfected animals have shown that it is possible to harvest prions in the test tube and recapitulate most of the biochemical and pathological events by the so-called PMCA (protein misfolding cyclic amplification) technology [4], [5]. Despite these great advances, understanding the molecular details of the protein conversion mechanism requires experimental setups relying on pure and defined components mimicking most of the features associated to prion formation. Experiments using exclusively recombinant PrP (recPrP) have so far failed to show infectivity in wild-type animals in a first passage [6], [7]. However, highly infectious synthetic prions have been reported using mixtures of recPrP, lipids and mouse-extracted RNA molecules submitted to PMCA [8]. RecPrP aggregates with low and heterogeneous infectivity were also achieved using modified PMCA experiments in reactions containing recPrP and mixtures of detergents (SDS and triton) [9], [10]. The lack of highly infectious material prepared with protein-only inoculates suggests that accessory co-factor molecules may be essential for prion infectivity in mammals [11]–[13].

Experimental approaches aimed to induce the conversion of recPrP into amyloid-like aggregates with some of the features associated to PrP^Sc^ have classically relied on the use of chemical and/or physical agents promoting partial or total protein denaturation such as guanidine hydrochloride, urea, SDS, temperature, pH, etc. [9], [14]–[21]. Most of these protocols yield recPrP aggregates resembling typical amyloid fibrils. This is in agreement with the current hypothesis that most, if not all, proteins have intrinsically the capability to be converted into amyloids [22]. Although PrP^Sc^ isolates exhibit some amyloid-like features, they rarely form classical amyloid fibrils [23], [24]. Thus it is unclear whether the formation of large amyloid aggregates is necessary for infectivity. RecPrP has been converted into PrP^Sc^-like aggregates when combined with lipids under physiological conditions and in the absence of denaturants [25]. Interestingly, as mentioned above, these same aggregates were later shown to be infectious in wild-type mice when RNA molecules were added to the mixture that was then subjected to PMCA cycles [8].

Salts have been previously used as a more physiological way of inducing protein misfolding and formation of amyloids [26]–[29]. It has been previously demonstrated that recPrP show a dual behavior in the presence of stabilizing salts, which is characterized by an initial destabilization at low concentrations followed by stabilizing effects at high concentrations according to the Hofmeister series [30], which is a classification of ions in order of their effect on protein solubility [31]. Sodium chloride can stimulate formation of recPrP amyloid in a concentration dependent manner under non-physiological conditions including very low pH and high temperatures [29]. Here, we tested the effect of kosmotropic/stabilizing salts on the misfolding pathway of full-length recPrP using strictly physiological temperature and pH. Our results show that kosmotropic anions specifically promote formation of PrP^Sc^-like aggregates in reactions containing only protein as the main organic molecule.

## Results

### Kosmotropic salts induce formation of protease-resistant aggregates of full length recPrP

**Figure 1:**
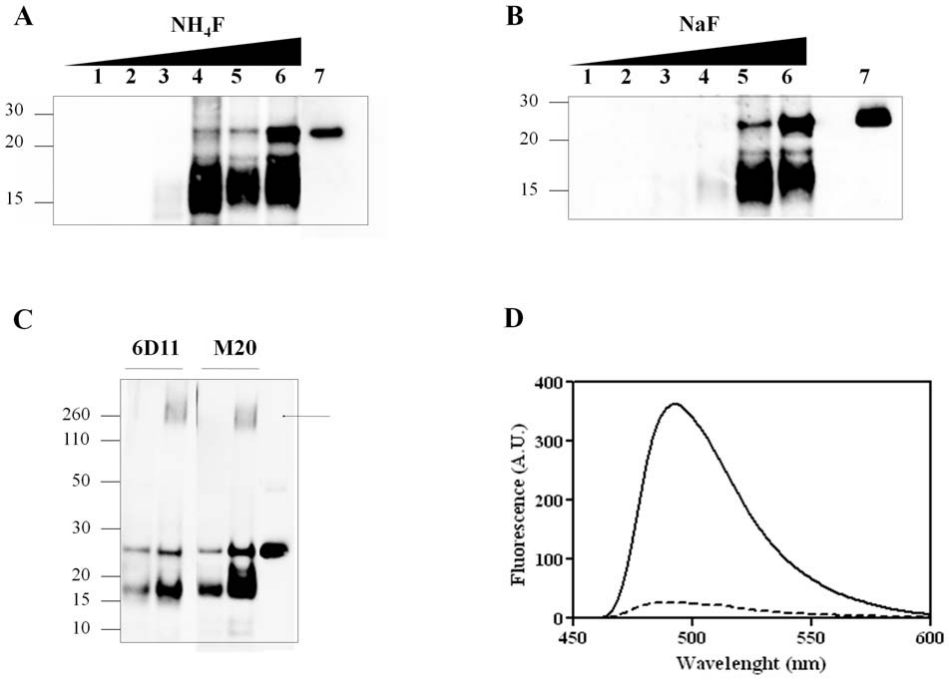
Formation of protease-resistance recPrP aggregates (recPrP^res^). ***A-B:*** RecPrP was incubated with different concentrations of NH_4_F (***A***) and NaF (***B***) as described in materials and methods, followed by Western Blotting. Salts concentrations (mM) for the reactions were 0, 100, 200, 300, 400 and 500 for lanes 1, 2, 3, 4, 5 and 6, respectively. A small amount of undigested recPrP used as a marker of electrophoretical migration is shown on lane 7 for each figure. ***C***: Antibody mapping analysis of protease resistance fragments was performed using a 400 mM NH_4_F-based reaction incubated for 24 hrs. Duplicated samples at two different dilutions (1/2 and 1/1 per left and right lane, respectively) were western-blotted using monoclonal antibodies 6D11 and M-20. The arrow indicates the presence of oligomeric species. ***D:*** RecPrP was incubated for 0 hrs (dashed line) or 24 hrs (solid line) with 400 mM NH_4_F and then fluorescence emission spectra of samples in the presence of 10 uM Th-T was recorded. An emission maximum was obtained at 491 nm when excited at 435 nm, typical of amyloid-like aggregates.

Prions composed of brain-derived PrP^Sc^ are known to be partially resistant to degradation by proteases [1]. We incubated recPrP in the presence of various salts to test the formation of protease-resistant material. In initial experiments, we found that the kosmotropic salt ammonium fluoride (NH_4_F) induced the formation of recPrP aggregates when incubated for 30 hrs using agitation/incubation cycles. Interestingly, these aggregates exhibited partially resistant to protease-degradation in a salt concentration-dependent manner as shown by Western Blotting analysis (Fig. 1A). The salt concentration at which protease-resistant recPrP (recPrP^res^) was first detected was around 300 mM. At lower concentrations we did not observe any protease-resistant material. The main degradation fragment exhibited a 16–17 kDa molecular weight, which is in agreement with the sizes reported for unglycosylated GPI-less PrP^Sc^ [32]. We also noticed that at high concentrations of salt, the signal of undigested recPrP also increased, which may be indicative of salting-out effects occurring during the reactions. By high speed ultracentrifugation, we determined that about sixty percent of the total protein is aggregated after a 24 hrs reaction, from which about twenty percent exhibited protease-resistance (data not shown). In order to determine whether the observed effect caused by NH_4_F was salt-specific, we used sodium fluoride (NaF), another known kosmotropic agent that promotes stabilization of structured states of most proteins, including recPrP at high salt concentrations [30]. Interestingly, under the same experimental conditions, NaF induced the formation of protease-resistant recPrP species in a concentration-dependent manner with similar sizes as those observed with NH_4_F (Fig. 1B).

PrP^Sc^ degradation occurs at the N-terminal region of PrP, implying that the protease-resistant material that retains infectivity properties is a C-terminal truncated portion of PrP [33]–[35]. In order to map the topology of the protease-resistant bands observed after incubation with NH_4_F, we used two different antibodies. mAB 6D11 is known to target a region between 93–109 within PrP of different species, including mouse. We also used the polyclonal antibody M-20 that recognizes an epitope near the C-terminus of PrP. All the protease-resistant fragments gave positive signal with both antibodies, suggesting that the products exhibit a similar mapping profile to that of PrP^Sc^, and that the 16–17 kDa species are the main proteolytic fragments upon digestion (Fig. 1C). Faints bands corresponding to 10–12 kDa molecular weight fragments were also detected with these antibodies.

Visible aggregation was evident at the end of the reaction in the presence of both kosmotropic salts. To rule that formation of unspecific recPrP aggregates may be taking place, we incubated these aggregates with the amyloid-specific dye thioflavin T (Th-T) [17], [36]. The Th-T signal significantly increased, suggesting formation of amyloid-like particles (Fig. 1D).

### The anion and not the cation is responsible for inducing protease-resistant recPrP

**Figure 2:**
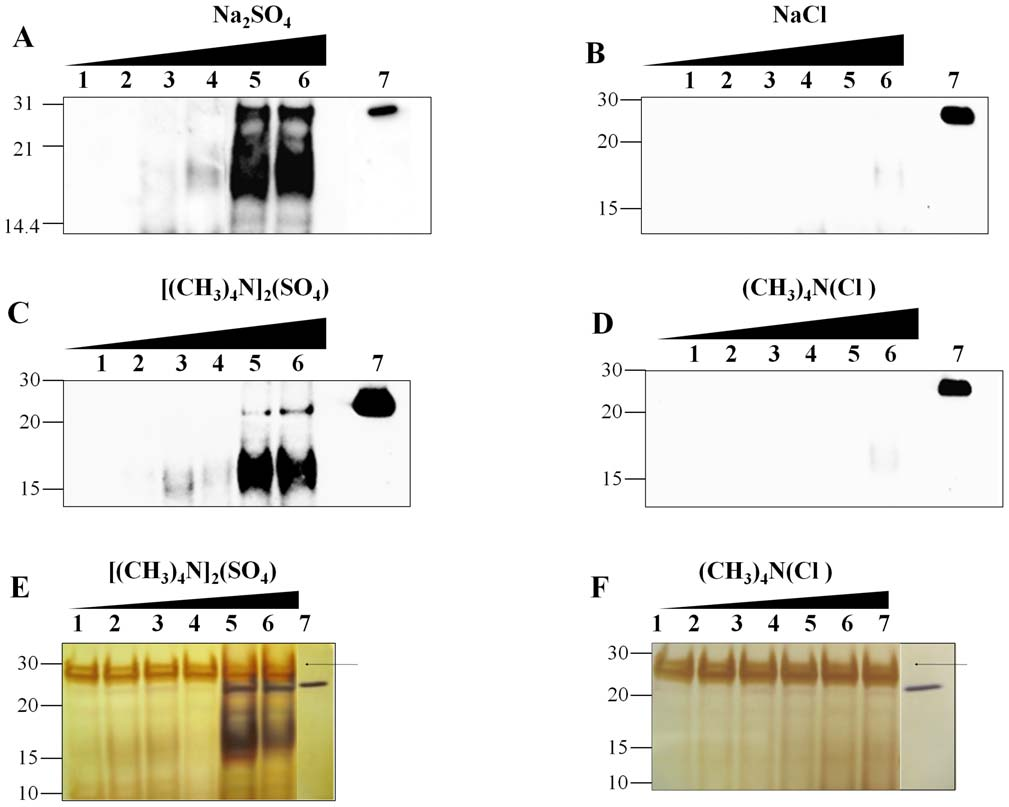
Kosmotropic anions induce formation of PrP^Sc^-like protease-resistant species. ***A–F:*** RecPrP was incubated with different concentrations of sodium sulfate (Na_2_SO_4_, ***A*** *)*, sodium chloride (NaCl, ***B***), tetramethylammonium sulfate (((CH_3_)_4_N)_2_(SO_4_), ***C,E***) and tetramethylammonium chloride ((CH_3_)_4_N(Cl), ***D,F***) as described in Experimental Procedures, followed by Western Blotting (***A–D***) or silver staining (***E*** **,** ***F***). Salts concentrations (mM) for the reactions were 0, 100, 200, 300, 400 and 500 for lanes 1, 2, 3, 4, 5 and 6, respectively. Undigested recPrP standard is shown on lane 7 for each figure. The arrows indicate the signal corresponding to proteainse K (PK).

We previously observed that incubation of recPrP in the presence of high concentrations of either NH_4_F or NaF led to the formation of protease-resistant species (Fig. 1A and B), suggesting that the kosmotropic cation ammonium is not essential for this phenomenon. In order to determine whether the effect observed is dependent on the anion or cation, we incubated recPrP in the presence of different salts which differed in their kosmotropicity as well as on the nature of the kosmotropic ion (Fig. 2). High concentrations of sodium sulfate (Na_2_SO_4_), which has a kosmotropic anion and a neutral cation produced a similar effect to those described in Fig. 1 (Fig. 2A). However, when recPrP was incubated at the same concentrations of sodium chloride (NaCl), no protease-resistant species were observed (Fig. 2B), suggesting that kosmotropic anions are essential in generating PrP^Sc^-like recPrP species. In order to further assess the specificity of kosmotropic anions on the observed effects, we incubated recPrP in the presence of the kosmotropic salt tetramethylammonium sulfate ((CH_3_)_4_N)_2_(SO_4_). Formation of protease-resistant recPrP was again observed at similar salt concentrations as those described previously (Fig. 2C). When the protein was incubated in the presence of tetramethylammonium chloride ((CH_3_)_4_N)_2_(Cl), which retains the kosmotropic cation (tetramethylammonium) but replaces sulfate by chloride anion, there was again no formation of protease-resistant species (Fig. 2D). These results provide strong support for a specific role of the kosmotropic anion. In order to rule out that formation of smaller or different protease-resistant species non-recognized by the monoclonal antibody 6D11 may be taking place in the reaction using ((CH_3_)_4_N)_2_(Cl), we analyzed the reaction products described in Fig. 2D by silver-staining. Again, we confirmed the absence of any detectable protease-resistant species (Fig. 2F). Interestingly, silver staining analysis of recPrP incubated in ((CH_3_)_4_N)_2_(SO_4_) yielded a main 16–17 kDa protease-resistant fragment (Fig. 2E) similar to that observed by western blots, and very faint bands corresponding to smaller 10–12 kDa fragments, similar to those observed with the C-terminal specific anti-PrP antibody (Fig. 1C). We did not observed fragments with molecular weight smaller than 10 kDa under any of the conditions tested (data not shown). To rule out an adverse effect of the salts in the proteolytic activity of PK, we used BSA as a control and confirmed that PK retains its proteolytic activity in the presence of kosmotropic salts even at the highest concentrations used in our studies (data not shown).

### Time-dependent formation of recPrP^res^

**Figure 3:**
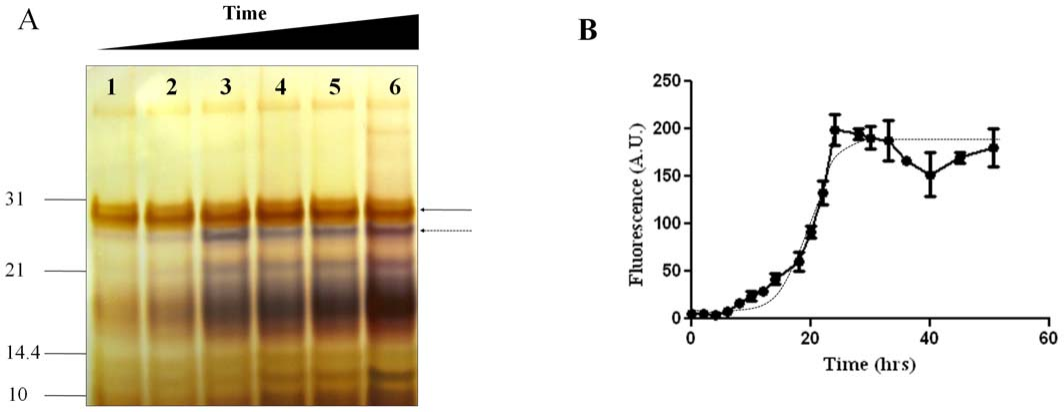
Time-dependent formation of recPrP^res^. ***A:*** RecPrP was aggregated in 400 mM NH_4_F for different times: 0 hrs (lane 1), 3 hrs (lane 2), 10 hrs (lane 3), 24 hrs (lane 4), 72 hrs (lane 5) and 144 hrs (lane 6) and the recPrP^res^ product was analyzed by silver staining. PK signal is highlighted by the solid arrow. The undigested recPrP signal is indicated by the dashed arrow. In all panels, samples were digested using PK at 1/10 PK/recPrP ratio for 1 hrs at 37°C and then subjected to silver staining. Molecular weights markers (kDa) are shown on the left side. ***B:*** A similar reaction was followed by the increase in Th-T signal in time. Each time point corresponds to the mean and standard error of 3 independent replicates. The points fit very well to a sigmoidal curve (dashed line).

A time-course experiment was performed in order to evaluate the progress of the reaction over time. We chose NH_4_F as the kosmotropic salt and followed the reaction for several days by silver staining analysis in order to provide a full spectrum of protease-resistant fragments. The formation of recPrP^res^ rapidly increased in the first hours of incubation and reached saturation after 4–6 days (Fig. 3A). We also noticed that the intensity of the lower molecular weight protease-resistant species (10–12 kDa) increased in time in a similar way as those of higher sizes, but were always a minor component. Moreover, the signal of the band associated to undigested full length recPrP also increased with a similar pattern. However, after several hours of reaction, this signal remained steady, while the protease-resistant digested products kept increasing, suggesting a more specific effect of the salt on the formation of the PrP^Sc^-like protease-resistant species.

To study the time dependency of amyloid formation and whether its kinetics followed a seeding-nucleation model typical of amyloids, we measured the Th-T signal as a function of time. The formation of Th-T positive aggregates exhibited a classical sigmoideal behavior with a lag-phase of around 15 hrs, followed by a exponential face, reaching a maximum at around 28 hrs (Fig. 3B) and then a small decay of the signal, probably due to formation of Th-T-inaccessible clumps of aggregates.

### Structural characterization of recPrP^res^ aggregates

**Figure 4:**
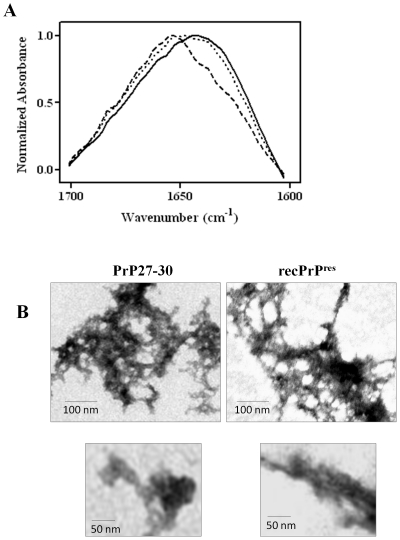
Structural properties of recPrP aggregates. ***A:*** Buffer and baseline-corrected FTIR spectra of PK-treated salt-induced recPrP aggregates (solid line) compared to those of soluble recPrP (dashed line) and PrP27-30 purified from the brain of mouse infected with RML prions (dotted line). FTIR spectra were obtained using the conditions described in Experimental Procedures. ***B:*** To study the morphology of the PK-treated recPrP aggregates, samples were loaded onto EM grids, stained with silver nitrate and visualized under TEM. Representative images for both PrP27-30 and recPrP^res^ aggregates are shown at two different magnifications (see the magnification bars).

To study the structural features of salt-induced recPrP^res^ aggregates, samples were analyzed by by FT-IR spectroscopy and compared it to soluble recPrP and brain-purified PrP27-30. Both recPrP^res^ aggregates and PrP27-30 showed a main absorbance peak at around 1639 cm^−1^ which is indicative of predominant beta-sheet secondary structure, while soluble recPrP exhibited high alpha-helical content, peaking at around 1658 cm^−1^ (Fig. 4A).

The ultra-structural morphology of protease-resistant recPrP aggregates (same as those used for FT-IR) assessed by TEM exhibited features similar to those observed with highly purified PrP27-30 obtained from the brain of prion infected animals (Fig. 4B). In both samples, we observed rod-shaped structures which formed large clumps of aggregates. Little or no classical amyloid fibrils were seen in any of the preparations. This is another indication that our protocol to induce misfolding of recPrP result in structures more similar to brain-derived PrP^Sc^ than protocols involving kaotropic agents which lead to the formation of classical amyloid fibrils [14]–[21].

### Citotoxicity of salt-induced recPrP^res^

**Figure 5:**
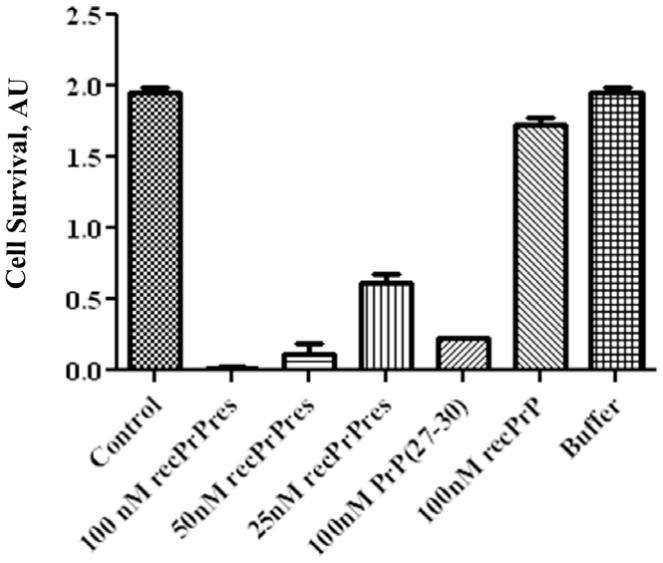
RecPrP^res^ aggregates are similarly neurotoxic as PrP27-30. RecPrP^res^ aggregates were produced by incubation for 24 hrs with 400 mM NaF followed by PK-digestion for 1 hrs at 37°C. 100, 50 and 25 nM of dialyzed recPrP^res^ aggregates were added to the medium of 1×10^5^ N2A neuroblastoma cells and cell viability was measured after 24 hrs of incubation using the MTT assay. As a negative control, the same volume of PBS was added to the well (control). Purified PrP27-30 from RML infected mice brain, soluble recPrP (recPrP) and the reaction buffer without protein (buffer) were also assayed as controls. All experiments were done in triplicate and the values correspond to the average ± standard error. The reduction of cell viability produced by addition of recPrP^res^ or PrP^Sc^ was highly significantly (P<0.001) different from soluble recPrP and the buffer control, as determined by student t-test.

We next sought to test whether the protease-resistant fragments generated in the presence of salts acquire toxic features against neuroblastoma cells. Initially we attempted performing the experiments adding directly the recPrP incubated with salts onto the cells. However, NaF *per se* was highly toxic to cells (data not shown). Thus, we first removed the salt by extensive dialysis before adding the proteins to the cell cultures. Under these conditions we observed high levels of toxicity of recPrP^res^ as measured by the MTT cell viability assay (Fig. 5). The citotoxic activity occurred at very low concentrations of recPrP^res^ comparable to those produced by PrP27-30 purified from the brain of prion infected animals. These concentrations are 1000-times lower than those used with small PrP fragments polymerized into amyloid fibrils [37], [38]. As expected, soluble recPrP and the dialysis buffer gave a near-to-zero toxicity (Fig. 5).

## Discussion

PrP misfolding is a central event in the pathogenesis of prion diseases. The mechanism responsible for the PrP^C^ to PrP^Sc^ conversion and the potential role of accessory molecules in this process are not completely understood [12], [39]. Several *in vitro* models have been proposed to study the mechanism of prion conversion. Among them extensive studies have been done using recPrP forced to aggregate into amyloid fibrils by using partially denaturing conditions, including chemical (guanidine hydrochloride, urea, SDS, etc.) and/or physical denaturants (temperature, pH, pressure, etc.) [14]–[21]. In some of these studies recPrP aggregates displayed various biochemical and even infectious properties of PrP^Sc^ [6], [7], [10], [17]. All of these experimental approaches rely on the assumption that partially unfolding states of the protein are directly involved in the amyloid formation pathway [22], [40]. In this work, we aimed to test whether recPrP can be folded into a PrP^Sc^-like conformation by more physiological conditions, and in the absence of any organic molecule, using exclusively stabilizing salts.

Protein-stabilizing salts have been classically shown to affect fibril formation in many amyloid systems [27], [29], [41]–[45]. In most cases, the lag phase of the process is accelerated at high concentrations of anions in an ion-specific manner that follows the Hofmeister series [26], [27], [29], [42]–[44], indicating a predominance of protein-water-ion interactions [43]. The Hofmeister series provides a general classification of ions which proceeds according to their effect on protein solubility [31]. Generally, ions are arranged from highly stabilizing (kosmotropic) to destabilizing (chaotropoic) following the order F−>PO_4_^−3^>SO_4_^−2^>Cl^−^>Br^−^>I^−^>CN^−^ for anions and (CH_3_)_4_N^+^>NH_4_^+^>K^+^>Na^+^>Li^+^>Mg^+2^ for cations, among others [31]. Aggregation of recPrP in the presence of the protein stability-wise neutral salt NaCl was reported at very low pHs and high temperatures [29]. In addition, recPrP stability at physiological conditions seems to follow the Hofmeister series order at high salt concentrations [30]. In this work, we found that at physiological pH and temperature, high concentrations of kosmotropic anions specifically promoted formation of protease-resistant aggregates that are reminiscent to those formed with bona-fide prions. The protease resistance fragments exhibited an approximated size of ∼16–17 kDa (Fig. 1), which is in agreement with sizes reported for PrP27–30 (subtracting the weights associated to GPI anchor and glycans) [1], [32]. Smaller fragments with molecular weights in the range of 10–12 kDa, reminiscent of the size of those observed in some TSEs cases [46]–[48] were also detected albeit at much lower quantity (Fig. 1C, 3A). Kosmotropic cations did not produce detectable protease-resistant fragments of any size (Fig. 2B, D and F). Interestingly, we observed that NH_4_F induced recPrP misfolding at lower salt concentrations compared to NaF, though the anion is the same in both cases (Fig. 1). The more stabilizing cation NH_4_^+^ may exert a synergistic effect on recPrP misfolding when coupled with kosmotropic anions. A similar but less clear effect was also observed with SO4 when coupled to (CH_3_)_4_N^+^ but not with Na^+^ (Fig. 2). More experiments are needed to clarify this. Although the salts used in this study are not considered denaturing agents, they may have some impact on the activity of the protease used in our studies. To rule out any significant effect of the salt on the protease activity, we did a control digestion assay of BSA in the presence of PK. We observed that PK degradation ability was not affected by the salt concentrations used in our assay (data not shown). This is not surprising considering that PK is completely active at high concentrations of NaCl [49]. Additional evidence to rule out an effect of the salt in protease activity comes from our time-course experiments. As showed in figure 3A at short times of incubation with salt, only a faint signal of protease-resistant recPrP is observed, despite the fact that the PK digestion conditions, including protease, PrP and salt concentrations, were the same as those at longer incubation times in which a high quantity of recPrP^res^ was observed.

The recPrP^res^ aggregates exhibited classical features associated to amyloids such as an increase in Th-T fluorescence emission, sigmoideal time-dependent aggregation mechanism and changes in secondary structure leading to higher contents of beta-sheet. We were not able to observe formation of typically well-defined amyloid fibers by TEM analysis. Classical amyloid fibrils are typically formed by using partially denaturing conditions, as it has been shown for recPrP in various reports [6], [14], [17], [18], [50], [51]. On the other hand, bona fide PrP^Sc^ isolated from the brains of prion infected wild-type animals usually exhibits relatively low content of amyloid fibrils (Fig. 4B) [23], [24]. Interestingly, the morphology of the aggregates consisting of clumps of rod-like structures was very similar between PrP^Sc^ and salt-induced recPrP^res^ (Fig. 4B). It is possible that kosmotropes-induced aggregates follow a different aggregation pathway than those formed under partially denaturant conditions, by for instance retaining some native-like structure. Amyloids generated under non-denaturing conditions have been previously shown to exhibit such features [40].

**Figure 6:**
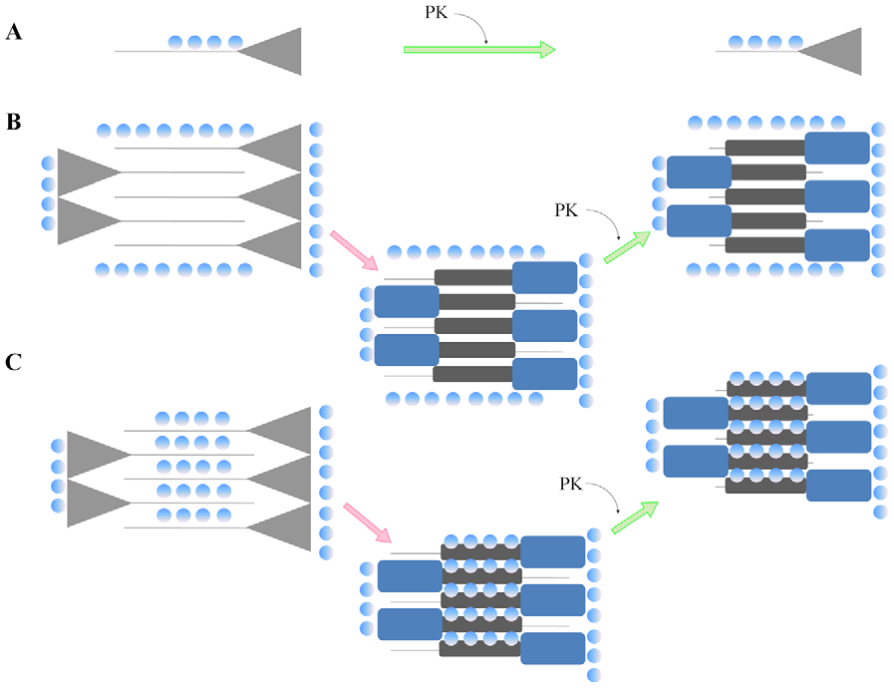
Potential mechanisms for recPrP aggregation induced by kosmotropic salts. The soluble monomeric recPrP is represented as a two-domain protein, C-terminal globular domain (triangle) containing a large proportion of alpha-helical structure and the natively-unfolded N-terminal domain (solid line). Misfolded recPrP acquires intermolecular beta-sheet secondary structure in the natively unfolded region 90–145 (represented as a dark gray horizontal rectangle). The structural fate of the C-terminal domain is unclear, but most likely involves a conformational rearrangement (represented as a dark blue rectangle). The kosmotropic anions are represented by the blue circles. The dotted arrows indicate a misfolding process, while the solid arrows represent the protease digestion reaction (PK). Three putative models to explain the effect of the salt on inducing the formation of recPrP^res^ are proposed. ***A:*** Binding of kosmotropic anions may occlude protease cleavage sites within the N-terminal domain of PrP. However, this model per se does not account for the partial resistance to proteolytic degradation of the whole C-terminal domain as well as for the structural changes induced by salt. ***B*** **:** A salting-out-like mechanism locally increases PrP concentration in a native-like conformation, followed by either an induction or acceleration of misfolding to form intermolecular beta-sheets giving rise to PrP^Sc^-like aggregates. ***C*** **:** A combined effect of high concentrations of kosmotropic anions that partially salt-out recPrP in a close-to-native fold from the bulk solution, along with specific anion binding to PrP that further stabilizes the N-terminal domain induce a protease-resistant recPrP conformation with PrP^Sc^ features.

The mechanism by which kosmotropic salts induce PrP^Sc^-like properties in recPrP is not known, but may involve a specific interaction with the protein promoting conformational changes (Fig. 6). Salts have been previously shown to be required by PrP^Sc^ in order to exhibit full protease-resistance features [49]. One potential scenario to explain recPrP partial resistance to proteloytic degradation observed upon incubation with kosmotropic anions may be simply due to electrostatic binding of ions to the highly basic N-terminal region blocking the access to cleavage sites by the protease (Fig. 6A). However we consider this scenario unlikely since chloride ion did not exert a significant effect (Fig. 2B, D and F). Moreover, positive charges are spread all over the N-terminal domain, which should cause an overall, unspecific protection against degradation. The observed protease resistance of the reminder C-terminal domain is also not accounted by this model. We also rule out a high specificity for the kosmotropic anion due to the high salts concentrations required to observe the protease-resistant bands and the fact that different kosmotropic anions produced similar results. Accordingly, stabilizing salts may promote PrP misfolding by increasing the local concentration of the protein through salting-out mechanisms (Fig. 6B). Under this scenario, folding intermediates or even PrP^Sc^-like particles originally present at very low concentrations would increase their effective concentration, accelerating the nucleation and seeding processes. As suggested elsewhere, fibril formation can be conceived as a specific case of salting-out in a similar way as general protein crystallization proceeds [43]. However, salting-out by itself may not be the only mechanism involved in generating PrP^Sc^-like aggregates, because kosmotropic cations were not able to promote protease-resistance (Fig. 2D, F) though visible aggregation was clear upon reactions at high salt concentrations (data not shown). Soluble recPrP is known to be thermodynamically stabilized at high concentrations of kosmotropic anions such as fluoride and sulfate, whereas chloride induces a rather destabilizing effect [30]. In our work, these two kosmotropic anions strongly stimulated formation of protease-resistant aggregates, whereas chloride, even when bound to a highly stabilizing cation, did not have a detectable effect. Sulfate and phosphate anions decorating the surface of certain macromolecules such as heparan-sulfate and polynucleotides have been proposed to play a role in PrP conversion [39], [52]–[54]. Interestingly, these macromolecules have also been proposed to serve as scaffolds that concentrate PrP molecules on its surface in addition to providing structure-compatible negatively charged groups, thereby acting as a potential catalyst for PrP^Sc^ formation [53], [55]–[58]. Our work is consistent with those reports, suggesting that increased local concentrations induced by interaction with kosmotropic anions may be critical for the conversion process. In addition, the acquisition of protease-resistance in part of the natively unfolded N-terminal domain may be a manifestation of disorder-to-order structural transitions within the region that are induced and/or stabilized by interaction with stabilizing anions (Fig. 6C). The electrostatic stabilization of the unstructured PrP region (residues ∼90–125) may be critical for misfolding due to its highly basic nature rather than in the globular C-terminal domain (residues ∼125–230) which is more neutral at pH 7 [59]. Upon conversion, the globular domain may undergo structural rearrangement through an independent pathway though this is still matter of debate. A recent report provides evidence for a major refolding within this region of PrP^Sc^ [60].

Our studies demonstrate that recPrP aggregates exhibiting PrP^Sc^-like biochemical properties can be readily formed using stabilizing salts. Synthetic infectious prions have been achieved using recPrP, lipids and mouse-derived RNA molecules [8]. Highly variable and low infectivity was also reported using recPrP in a mixture of detergents and saline buffer [10]). The formation of prions made out of strictly protein in buffer without organic additives has not been achieved so far. Our findings present a system in which recPrP misfolding with features associated to PrP^Sc^ can be induced only by varying the concentration of kosmotropic anions in a close-to-physiological experimental setup. Infectivity studies with these samples are currently ongoing. If these studies show the generation of infectivity, salts may represent the simplest “co-factor” molecule required for prion generation.

## Materials and Methods

### Full length recombinant mouse PrP (recPrP) cloning, expression and purification

DNA primers (forward 5′-CTGTCTAGAATGAAAAAGCGGCCAAAGCCTGG-3′ and reverse 5′ GAGCTCGAGTTAGGATCTTCTCCCGTCGTAATA 3′) with restriction sites for Xba I (forward primer) and Xho I (reverse primer) were synthesized and used to amplify by PCR the murine *prnp* gene 23–230 from C57 mouse genomic DNA extracted from the animals tail. The PCR product was digested with Xba I and Xho I restriction enzymes (New England BioLabs) and ligated into previously Xba I and Xho I – digested pET303/CT-His (Invitrogen®) plasmid. Plasmid DNA production was then performed using E. coli DH10B-T1 cells (Invitrogen®). For expression, freshly transformed cells (E. coli BL21 Star(DE3) cells, Invitrogen®) were grown in 5 ml of Terrific Broth medium supplemented with carbenicillin (100 ug/ml) at 37°C for 6 h. The starter culture was then diluted into 50 ml of the same medium and grown for another 6 h. This culture was finally diluted into 750 ml of the same medium and grown until it reached 0.7 OD. One millimolar IPTG (isopropyl β-D-thiogalactopyranoside) was then added, and the cells were induced for 5 h. The culture was harvested by centrifugation and stored at −80°C. For purification, cell pellets were thawed and resuspended in buffer A (50 mM Tris-HCl, pH 8.0, 1 mM EDTA, and 100 mM NaCl). Cells were lysed by adding 0.5 mg/ml lysozyme and subsequently sonicated. The released inclusion bodies were pelleted by centrifugation at 22,000 g and then washed twice with buffer A supplemented with 0.05% (v/v) Triton X-100. The inclusion bodies containing recPrP were solubilized for 2 hrs with buffer B (10 mM Tris-HCl, 100 mM Na2HPO4, pH 8.0, 100 mM NaCl, 10 mM -mercaptoethanol, 6 M GdnCl) and then purified by using standard immobilized metal affinity chromatography procedure. Briefly, the sample was bound to Ni Sepharose 6 Fast Flow resin (GE Healthcare) in batch mode for 1 hrs at room temperature and then washed with buffer B. RecPrP was on-column refolded for 6 hrs and eluted with buffer B supplemented with 500 mM imidazole and without GdnCl. The main peak was collected and quickly filtered to remove aggregates. The sample was buffer exchanged using Zeba desalting columns (Pierce®), further concentrated to ∼0.5 mg/ml, and flash-frozen at −80°C. Protease inhibitors (Complete® protease inhibitor mixture from Roche) were used throughout the purification to minimize degradation. The protein was confirmed to be monomeric and folded by SDS-PAGE, Western blotting, and circular dichroism. The concentration of recPrP was estimated by spectrophotometry using the BCA kit following manufacturer's specifications and confirmed by amino acid analysis.

### Brain-derived PrP^Sc^ purification

The protease-resistant core of PrP^Sc^ (PrP27-30) used in this work was obtained from RML-infected mouse brains and was purified as follows: 10% brain homogenates containing 10% sarkosyl were loaded onto a 20% sucrose cushion and centrifuged at 150,000× g for 3 hrs. The pellet was resuspended in Z3-14 zwitterion detergent, then loaded onto 20% sucrose cushion and centrifuged at 150,000× g for 3 hrs. This step was repeated twice. Pellet was finally resuspended in 1X PBS buffer and washed twice using the same 20% sucrose cushion. The PBS-resuspended pellet was further digested by 50 mg/mL PK for 2 hrs at 37°C and then collected by ultracentrifugation at 100.000× g for 30 hrs. This last step was repeated twice. The resulting PrP27-30 sample was shown to be highly pure (>90%) as assessed by silver staining and western blotting. PrP27-30 concentration was estimated by Western Blotting compared to known concentrations of soluble recPrP.

### Aggregation assays

All reactions were carried out in 0.5 mL Lo-Bind Eppendorf tubes (protease/nuclease free grade and further autoclaved). All reagents were of highest available quality and protease-free grade when available. Protease-free nanopure water was used to prepare all master solutions and reactions. Thawing of recPrP was followed by filtration through 100 kDa-cutoff Microcon tubes (Millipore) to remove possible aggregates formed upon thawing. Protein concentration was re-estimated upon filtration by spectrophotometry. RecPrP (0.1 mg/mL final concentration) was mixed with 40 mM Hepes pH 7.2 and different concentrations of salts in 100 uL final volume reactions. pH was re-adjusted upon salt addition. Aggregation experiments were conducted using a protocol adapted from a previous report [61]. Unless specified, reactions were placed in an Eppendorf Thermomixer for 24 hrs at 37°C with cycles of 1 minute agitation at 1500 rpm and 1 minute incubation. Samples were then treated with Proteinase K (PK) at a 1/10 molar ratio (PK/recPrP) for 1 hrs at 37°C in a water bath, unless specified differently. Analysis of digestion assays was performed by SDS-PAGE followed by Western blotting and silver staining. Antibodies 6D11 and M-20 were used for the analysis. To determine the amount of aggregated protein, a 24 hrs reaction was centrifuged at 30000× g for 30 min at 4°C. The pellet was resuspended in 6 M GdmCl and boiled for 10 min at 95°C. Protein concentration was estimated by spectrophotometry.

### Thioflavin-T binding assay

Highly pure thioflavin-T (Th-T) dye stock solution was added directly to reactions to a final concentration of 10 µM in black-walled 96-well plates. Fluorescence measurements of the samples were taken using a Microplate Reader Accessory adapted to a Hitachi Fluorescence Spectrophotometer F-7000, using an excitation wavelength of 435 nm and recording in the emission range between 400 and 600 nm. A scan speed of 240 nm/min was used as standard for all readings. To reduce interference noise from excitation light, a 475 nm emission cutoff filter was used.

### Fourier-Transform Infrared spectroscopy (FTIR)

FTIR experiments were conducted in an FT/IR-4100 spectrometer from JASCO. Monomeric recPrP was directly used as control sample. RecPrP aggregates were formed after a 24 hrs reactions in the presence of 400 mM NH_4_F, as described previously. Before measurements, recPrP aggregates were PK-treated at 37°C for 1 hr at 1/10 PK/recPrP ratio. The reaction was stopped by adding 5 mM Pefabloc (Roche®) and then concentrated by ultracentrifugation at 150,000× g for 1 hrs at 4°C. Protein slurry was then added on top of a diamond PRO450-S Attenuated Total Reflectance unit from JASCO adapted to the FT/IR-4100 system. System parameters included 4.0 cm^−1^ resolution and an accumulation of 64 scans per sample. Data fitting and secondary structure calculations of samples were analyzed by multi-component analysis through the Secondary Structure Estimation (SSE) software from JASCO. Each sample measurement was optimized until the corrected experimental data and the fitting curve gave minimum absorbance differences.

### Negative staining Transmission Electron Microscopy (TEM)

The same recPrP aggregates and PrP27-30 used for FT-IR analysis were directly put onto FORMVAR–coated copper grids, air-dried for 5 minutes followed by filter-paper-drying of reaction excess. Grids with samples were negatively stained with 1% uranyl acetate for 1 min, wick off excess and dry for another 5 min at 70°C. Imaging was performed on a JEOL 1200 transmission electron microscope at 60 kV and captured by 1 k×1 k Gatan BioScan 792 CCD camera.

### Cell Viability Assay

N2A cells were cultured in DMEM supplemented with 10% fetal calf serum and antibiotics (10,000 U/ml Penicillin, 10 mg/ml streptomycin), at 37°C and 5% CO_2_. For cell viability analysis, cells were grown in collagen IV coated 96-well plates for 48 hrs in cell culture medium containing 1% serum before addition of recPrP aggregates and control reactions. Cell viability was quantified using 3-(4,5-dimethylthazol-2-yl)-5-3-carboxymethoxy- phenyl)-2-(4-sulfophenyl)-2H-tetrazolium (MTS) and phenazine methosulfate (PMS) according to the recommendations of the supplier (Roche).
